# GIGANTEA-ENHANCED EM LEVEL complex initiates drought escape response via dual function of ABA synthesis and flowering promotion

**DOI:** 10.1080/15592324.2023.2180056

**Published:** 2023-02-22

**Authors:** Zein Eddin Bader, Min Jae Bae, Akhtar Ali, Junghoon Park, Dongwon Baek, Dae-Jin Yun

**Affiliations:** a Department of Biomedical Science and Engineering, Konkuk University, Seoul, Republic of Korea; b Institute of Global Disease Control, Konkuk University, Seoul, Republic of Korea; c Plant Molecular Biology and Biotechnology Research Center, Gyeongsang National University, Jinju, Republic of Korea

**Keywords:** GIGANTEA, EEL, ABA biosynthesis, NCED3, circadian clock, drought escape

## Abstract

Plants use the regulation of their circadian clock to adapt to daily environmental challenges, particularly water scarcity. During drought, plants accelerate flowering through a process called drought escape (DE) response, which is promoted by the circadian clock component GIGANTEA (GI). GI up-regulates the flowering inducer gene FLOWERING LOCUS T (FT). Phytohormone Abscisic acid (ABA) is also required for drought escape, and both GIGANTEA and Abscisic acid are interdependent in the transition. Recent research has revealed a new mechanism by which GIGANTEA and the protein ENHANCED EM LEVEL form a heterodimer complex that turns on ABA biosynthesis during drought stress by regulating the transcription of 9-CIS-EPOXYCAROTENOID DIOXYGENASE 3 (NCED3). This highlights the close connection between the circadian clock and ABA regulation and reveals a new adaptive strategy for plants to cope with drought and initiates the DE response.

## Introduction

**Figure 1.**
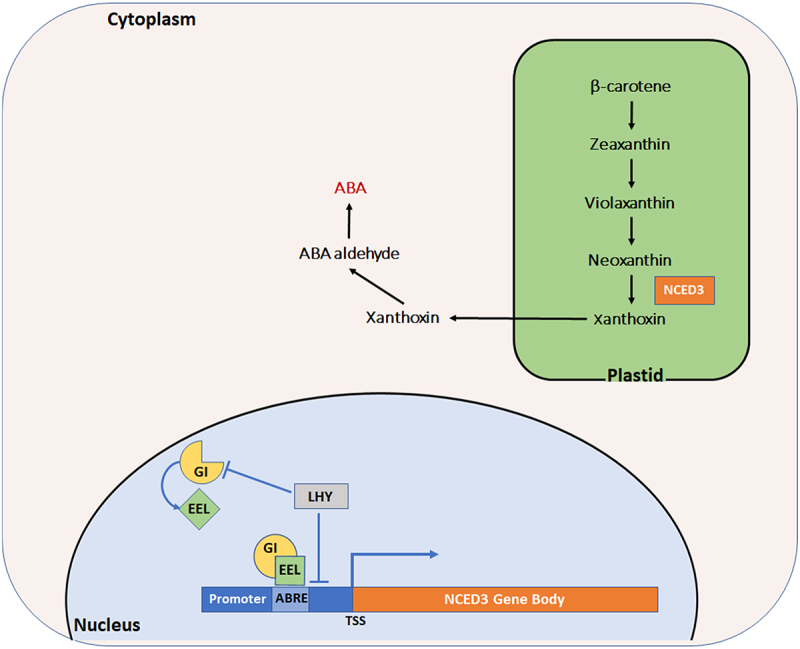
Schematic diagram showing GI-EEL regulation of ABA biosynthesis pathway.

Physiological and developmental plasticity in plants occurs at every level of complexity to cope with environmental stressors.^1^ Understanding the molecular, cellular and behavioral plant response mechanisms for adaptation to environmental challenges is crucial for solving world food insecurity problems and increasing global crop yield, ^[1,2]^ Circadian biology plays a crucial role in stress signaling in plants by coordinating the expression of genes involved in the response to stress conditions.^3^ The central component of the plant circadian clock is a set of genes known as clock genes, which form three interconnected loops known as the morning, central, and evening loops. The morning loop consists of the genes LHY (LATE ELONGATED HYPOCOTYL) and CCA1 (CIRCADIAN CLOCK-ASSOCIATED 1), expressed in the morning, promoting the expression of TOC1 (TIMING OF CAB EXPRESSION 1), a central component of the circadian rhythm. The central loop, composed of TOC1, PRR (PSEUDORESPONSE REGULATOR) genes, and LUX (LIGHT-REGULATED) genes, controls the expression of the evening loop genes and its own expression, forming a feedback loop to maintain the circadian rhythm. The evening loop, consisting of GI (GIGANTEA), ELF3 (EARLY FLOWERING 3), and ELF4 (EARLY FLOWERING 4), expressed in the evening, regulates LHY and CCA1 expression, which in turn regulate TOC1 expression. These interlinked circadian loops work together to generate a rhythmic pattern of gene expression, driving the circadian rhythm.^4–7^ Approximately 30% of the plant transcriptome, including transcripts involved in hormone biosynthesis pathways,^8–10^ shows diurnal expression and is regulated by circadian oscillation.^3^ In particular, abscisic acid (ABA), a phytohormone with a diurnal biosynthesis pattern and accumulation, regulates various physiological processes including seed dormancy, seed germination, post-germination seedling growth, abscission acceleration, and stomatal movement.^11^ Although various studies explain the link between ABA (biosynthesis, accumulation, and signaling) and circadian rhythm, the underlying mechanisms are still unclear.^8,12^ Adams *et al*. (2018) suggested that LHY, a core circadian clock transcription factor, plays a complex role in regulating the expression of a rate-limiting enzyme in the ABA biosynthesis pathway.^8^ In addition to LHY, other clock genes like TOC1, PRRs and GI also take part in the rhythmic accumulation of ABA through the indirect regulation of the key enzymes in ABA biosynthesis.^8,13,14^ The initial steps of ABA synthesis take place in plastids, where the carotenoids are converted to xanthoxin in a series of reactions by 9-cis-epoxycarotenoid dioxygenase (NCED), a rate-limiting enzyme family in ABA biosynthesis.^11^ Xanthoxin is relocated to the cytoplasm, where it is converted to active ABA by two catalytic reactions.^11^ The *NCED3* transcript is the most expressed among the NCED enzymes in plant stems and roots.^15^ NCED3 exhibits a diurnal transcription oscillation matching the diurnal pattern of ABA that peaks during daytime and declines at night.^13^ We have recently shown that ENHANCED EM LEVEL (EEL), a bZIP transcription factor, and GI binds to the ABRE motif of the *NCED3* promoter and promotes its transcription.^13^ These findings could explain the regulation of ABA biosynthesis by the circadian clock components that transcriptionally regulate the key enzymes of this process^13^ (Figure 1).

In the nucleus, GI forms a complex with EEL on NCED3 promoter bearing ABRE motif; this positively regulates NCED3 expression. In plastids, NCED3 enzyme is responsible for converting neoxanthin to xanthoxin, which relocates to the cytoplasm and undergoes catalytic reactions to form ABA.

## GI, a multifunctional protein, regulates plant development and stress response

**Figure 2.**
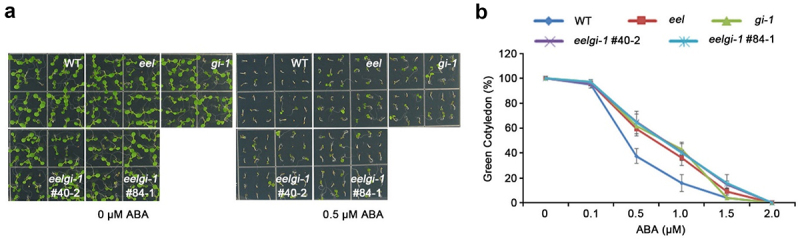
*Eel, gi-1*, and *eel/gi-1* double mutants show insensitivity toward ABA.

Over the last two decades, GI has received significant attention from researchers due to its multifunctional characteristics. GI regulates several key physiological processes including plant growth and development and plant responses to environmental stresses, such as salt, drought, cold and oxidative stress.^13,15–17^ Unlike other circadian clock proteins, GI has no DNA binding domain, but it modulates the plant transcriptome through interaction with other transcription factors.^18–21^ GI interactions with other proteins are versatile. On the one hand, GI binds to ZEITLUPE (ZTL) and acts as a co-chaperone protein that facilitates ZTL maturation and stability;^18^ on the other hand, it interacts with FLAVIN-BINDING, KELCH REPEAT, and F-BOX 1 (FKF1) to promote its ubiquitination function.^21^ GI plays a crucial role in regulating the temporal expression of CONSTANT (CO) protein in the nucleus under long-day conditions. The expression of CO is characterized by bimodal peaks in the early morning and late afternoon. ZTL plays a role in mediating the degradation of CO in the morning through direct binding to it. However, the expression of FKF1 and GI in the afternoon disrupts this process. The proteins form an active complex, with GI preferentially interacting with ZTL and inactivating its function. This leads to sequestration of CO from ZTL. Meanwhile, FKF1 stabilizes CO through forming a protein complex with it. ZTL also interacts with FKF1, inhibiting the FKF1-mediated CO stabilization, leading to destabilization of CO. GI’s preferential binding to ZTL also interferes with the complex formation between FKF1 and ZTL. These complex and sophisticated regulatory mechanisms allow for the highly accumulated expression of CO in the late afternoon of long days to control FLOWERING LOCUS T (FT) transcription.^21–23^ Although GI has contradictory molecular functions, it has a consistent physiological function in regulating the photoperiodic flowering pathway through modulating the florigen genes expression.^24,25^ Baek and colleagues (2020) explained the mechanistic regulation of ABA biosynthesis and accumulation by GI that seems inconsistent with the occurrence of flowering inhibition by exogenous ABA treatment,^13,26^ as the application of exogenous ABA to Arabidopsis thaliana plants significantly delays their floral transition.^26^ However, an increased level of endogenous ABA was observed during flowering in short-term drought stress in Arabidopsis.^27^ This phenomenon is known as the drought escape (DE) response.^28^ Recent reports have indicated the importance of GI in DE response via regulating ABA biosynthesis, suggesting that GI is involved in flowering promotion by both ABA synthesis and florigen genes regulation in response to drought stress.^13,27^ In contradiction, recent research article suggests that the highest accumulation of GI at noon plays a crucial role in establishing a phase of decreased ABA concentration and is associated with a negative regulation of ABA transcriptional responses and sensitivity.^29^ Thus, here we show unlike the wild-type plants, the loss-of-function mutant of *GI* (*gi-1*) exhibits an ABA insensitive phenotype (Figure 2); and this is additional evidence for the less endogenous ABA concentration in *gi-1* measured previously in *Beak et al*. and explains the close link between GI and ABA. In summary, upon binding to FKF1 and ZTL, GI regulates flowering, while its association with EEL promotes ABA biosynthesis and DE responses^13,21^ (Figure 1).

(A) Seeds of WT (Col-0), eel (SALK_021965), gi-1,^23^ and eel/gi-1 double mutants were germinated on 1/2 MS medium (1.5% [w/v] Suc, 0.6% [w/v] agar, pH 5.7) at 23°C with or without 0.5 μM ABA in a long-day chamber at 22°C. Photographs were taken 5 days after germination. (B) Green cotyledon rate was scored for different concentrations of ABA at 5 days. Error bars represent means ± SD of 36 seeds from three independent experiments.

## EEL, a bZIP type transcription factor, regulates ABA biosynthesis

**Figure 3.**
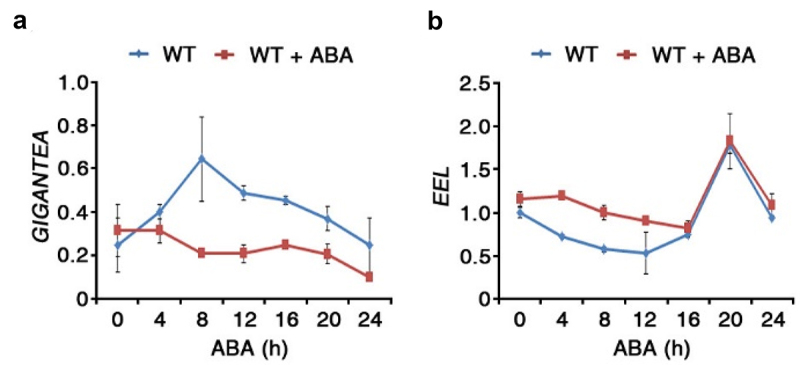
Expression response of *GI* and *EEL* to ABA stress response.

EEL is an ABI5/AtDPBF family member of the bZIP transcription factors in *Arabidopsis* and is involved in ABA signaling.^30,31^ The ABI5/AtDPBF proteins including EEL display significant localization in the embryo during the maturation phase.^30,32^ Accordingly, this could be additional evidence for the importance of EEL as a transcriptional regulator for the ABA-dependent stress signals during embryogenesis and seed maturation. Moreover, the transcriptional function of EEL pivots on forming either a homodimer or a heterodimer complex with other proteins.^13,30^ GI-EEL, a recently discovered heterodimer, positively regulates ABA synthesis in drought stress.^13^ Previous research attempted to address the fundamental question of how ABA signals are integrated into the photoperiodic flowering network. It provided evidence for ABA’s control of FT gene expression under normal and drought stress conditions by impacting photoperiodic signaling through GI. It also highlighted ABA’s negative effect on the floral transition of Arabidopsis that is independent of the photoperiodic pathway.^33^ However, our study reveals a different type of ABA regulation by the photoperiodic clock genes GI and EEL. As it is commonly observed that mutants with lower endogenous ABA content display an ABA-insensitive phenotype,^34–38^ it is interesting to note that the loss-of-function EEL mutant, along with the gi-1 mutant and the eel/gi-1 double mutant, also exhibit ABA-insensitive phenotypes (Figure 2). These phenotypes could be explained by the low level of endogenous ABA in these mutants due to the lack of ABA biosynthesis, along with the dual desensitization and degradation response toward exogenous ABA.^39^ In addition, it was observed that the expression of both *GI* and *EEL* is significantly reduced by exogenous ABA (Figure 3), which can be related to a negative feedback regulation of the increased exogenous ABA treatment. The reduced significant difference in the transcript levels after 16 h treatment (Figure 3) could be related to the ABA desensitization and degradation processes. This accumulated evidence strongly supports the critical role of the GI-EEL complex in manipulating ABA response.

Transcript levels of *GI* (A) and *EEL* (B) expressed in 10-day-old WT seedlings germinated in 1/2 MS medium sprayed with 50 μM ABA or Mock for indicated time points. *TUBULIN2* was used as an internal control for normalization. Bars represent means ± SD of three biological replicates with three technical replicates each. RNA extraction, cDNA synthesis, qRT condition and primer used in this report as indicated in Baek *et al*. ^13^

## Conclusion

In conclusion, despite conflicting research on ABA regulation of the floral transition under stress conditions,^40^ the role of the GI-EEL complex in regulating the diurnal oscillation of ABA biosynthesis is clear. This complex enhances the transcription of NCED3 by binding to its promoter, thus regulating the DE response. GI, a well-known regulator of the circadian clock and photoperiodic flowering, and EEL play a crucial role in helping plants adapt to short-term water shortages by regulating endogenous ABA levels. This highlights the interplay between phytohormones, particularly ABA, and the circadian clock as a key adaptive strategy for plants facing environmental challenges.

## References

[1] Cramer GR, Urano K, Delrot S, Pezzotti M, Shinozaki K. Effects of abiotic stress on plants: a systems biology perspective. BMC Plant Biol. 2011;11(1):163. doi:10.1186/1471-2229-11-163. 22094046 PMC3252258

[2] Ali A, Kim JK, Jan M, Khan HA, Khan IU, Shen M, Park J, Lim CJ, Hussain S, Baek D, et al. Rheostatic control of ABA signaling through HOS15-mediated OST1 degradation. Mol Plant. 2019;12(11):1447–5. doi:10.1016/j.molp.2019.08.005. 31491477

[3] Blair EJ, Bonnot T, Hummel M, Hay E, Marzolino JM, Quijada IA, Nagel DH. Contribution of time of day and the circadian clock to the heat stress responsive transcriptome in Arabidopsis. Sci Rep. 2019;9(1):4814. doi:10.1038/s41598-019-41234-w. 30886204 PMC6423321

[4] Cha JY, Kim J, Jeong SY, Shin G-I, Ji MG, Hwang J-W, Khaleda L, Liao X, Ahn G, Park H-J, et al. The Na+/H+antiporter SALT OVERLY SENSITIVE 1 regulates salt compensation of circadian rhythms by stabilizing GIGANTEA in Arabidopsis. Proc Natl Acad Sci U S A. 2022;119(33):e2207275119. doi:10.1073/pnas.2207275119. 35939685 PMC9388102

[5] Kim JA, Kim HS, Choi SH, Jang JY, Jeong MJ, Lee SI. The importance of the circadian clock in regulating plant metabolism. Int J Mol Sci. 2017;18(12): 2680. doi:10.3390/ijms18122680. 29232921 PMC5751282

[6] Phan KAT, Paeng SK, Chae HB, Park JH, Lee ES, Wi SD, Bae SB, Kim MG, Yun D-J, Kim W-Y, et al. Universal stress protein regulates the circadian rhythm of central oscillator genes in Arabidopsis. FEBS Lett. 2022;596(15):1871–1880. doi:10.1002/1873-3468.14410. 35644867

[7] Seo PJ, Mas P. STRESSing the role of the plant circadian clock. Trends Plant Sci. 2015;20(4):230–237. doi:10.1016/j.tplants.2015.01.001. 25631123

[8] Adams S, Grundy J, Veflingstad SR, Dyer NP, Hannah MA, Ott S, Carré IA. Circadian control of abscisic acid biosynthesis and signalling pathways revealed by genome-wide analysis of LHY binding targets. New Phytol. 2018;220(3):893–907. doi:10.1111/nph.15415. 30191576

[9] Covington MF, Harmer SL, Weigel D. The circadian clock regulates auxin signaling and responses in Arabidopsis. PLoS Biol. 2007;5(8):e222. doi:10.1371/journal.pbio.0050222. 17683202 PMC1939880

[10] Nitschke S, Cortleven A, Iven T, Feussner I, Havaux M, Riefler M, Schmülling T. Circadian stress regimes affect the circadian clock and cause jasmonic acid-dependent cell death in cytokinin-deficient Arabidopsis plants. Plant Cell. 2016;28(7):1616–1639. doi:10.1105/tpc.16.00016. 27354555 PMC4981127

[11] Chen K, Li G-J, Bressan RA, Song C-P, Zhu J-K, Zhao Y. Abscisic acid dynamics, signaling, and functions in plants. J Integr Plant Biol. 2020;62(1):25–54. doi:10.1111/jipb.12899. 31850654

[12] Seung D, Risopatron JPM, Jones BJ, Marc J. Circadian clock-dependent gating in ABA signalling networks. Protoplasma. 2012;249(3):445–457. doi:10.1007/s00709-011-0304-3. 21773710

[13] Baek D, Kim W-Y, Cha J-Y, Park HJ, Shin G, Park J, Lim CJ, Chun HJ, Li N, Kim DH, et al. The GIGANTEA-ENHANCED EM LEVEL complex enhances drought tolerance via regulation of abscisic acid synthesis. Plant Physiol. 2020;184(1):443–458. doi:10.1104/pp.20.00779. 32690755 PMC7479899

[14] Liu T, Carlsson J, Takeuchi T, Newton L, Farré EM. Direct regulation of abiotic responses by the Arabidopsis circadian clock component PRR7. Plant J. 2013;76(1):101–114. doi:10.1111/tpj.12276. 23808423

[15] Tan BC, Joseph LM, Deng WT, Liu L, Li QB, Cline K, McCarty DR. Molecular characterization of the Arabidopsis 9-cis epoxycarotenoid dioxygenase gene family. Plant J. 2003;35(1):44–56. doi:10.1046/j.1365-313x.2003.01786.x. 12834401

[16] Cao S, Jiang S, Zhang R. The role of GIGANTEA gene in mediating the oxidative stress response and in Arabidopsis. Plant Growth Regul. 2006;48(3):261–270. doi:10.1007/s10725-006-0012-8.

[17] Kim WY, Ali Z, Park HJ, Park SJ, Cha JY, Perez-Hormaeche J, Quintero FJ, Shin G, Kim MR, Qiang Z, et al. Release of SOS2 kinase from sequestration with GIGANTEA determines salt tolerance in Arabidopsis. Nat Commun. 2013;4:1352. doi:10.1038/ncomms2357. 23322040

[18] Cha JY, Kim J, Kim T-S, Zeng Q, Wang L, Lee SY, Kim W-Y, Somers DE. GIGANTEA is a co-chaperone which facilitates maturation of ZEITLUPE in the Arabidopsis circadian clock. Nat Commun. 2017;8(1):3. doi:10.1038/s41467-016-0014-9. 28232745 PMC5431898

[19] Ito S, Song YH, Imaizumi T. LOV domain-containing F-box proteins: light-dependent protein degradation modules in Arabidopsis. Mol Plant. 2012;5(3):573–582. doi:10.1093/mp/sss013. 22402262 PMC3355347

[20] Kubota A, Ito S, Shim JS, Johnson RS, Song YH, Breton G, Goralogia GS, Kwon MS, Laboy Cintrón D, Koyama T, et al. TCP4-dependent induction of CONSTANS transcription requires GIGANTEA in photoperiodic flowering in Arabidopsis. PLoS Genet. 2017;13(6):e1006856. doi:10.1371/journal.pgen.1006856. 28628608 PMC5495492

[21] Song YH, Smith RW, To BJ, Millar AJ, Imaizumi T. FKF1 conveys timing information for CONSTANS stabilization in photoperiodic flowering. Science. 2012;336(6084):1045–1049. doi:10.1126/science.1219644. 22628657 PMC3737243

[22] Hwang DY, Park S, Lee S, Lee SS, Imaizumi T, Song YH. GIGANTEA regulates the timing stabilization of CONSTANS by altering the interaction between FKF1 and ZEITLUPE. Mol Cells. 2019;42(10):693–701. doi:10.14348/molcells.2019.0199. 31617339 PMC6821452

[23] Kim WY, Fujiwara S, Suh -S-S, Kim J, Kim Y, Han L, David K, Putterill J, Nam HG, Somers DE, et al. ZEITLUPE is a circadian photoreceptor stabilized by GIGANTEA in blue light. Nature. 2007;449(7160):356–360. doi:10.1038/nature06132. 17704763

[24] Jose J, Bánfalvi Z. The role of GIGANTEA in flowering and abiotic stress adaptation in plants. COLUMELLA – J Agri Environ Sci. 2019;6(1):7–18. doi:10.18380/SZIE.COLUM.2019.6.1.7.

[25] Abdul-Awal SM, Chen J, Xin Z, Harmon FG. A sorghum gigantea mutant attenuates florigen gene expression and delays flowering time. Plant Direct. 2020;4(11):e00281. doi:10.1002/pld3.281. 33210074 PMC7665845

[26] Wang Y, Li L, Ye T, Lu Y, Chen X, Wu Y. The inhibitory effect of ABA on floral transition is mediated by ABI5 in Arabidopsis. J Exp Bot. 2013;64(2):675–684. doi:10.1093/jxb/ers361. 23307919 PMC3542054

[27] Riboni M, Galbiati M, Tonelli C, Conti L. GIGANTEA enables drought escape response via abscisic acid-dependent activation of the florigens and SUPPRESSOR OF OVEREXPRESSION OF CONSTANS. Plant Physiol. 2013;162(3):1706–1719. doi:10.1104/pp.113.217729. 23719890 PMC3707542

[28] Shavrukov Y, Kurishbayev A, Jatayev S, Shvidchenko V, Zotova L, Koekemoer F, de Groot S, Soole K, Langridge P. Early flowering as a drought escape mechanism in plants: how can it aid wheat production? Front Plant Sci. 2017;8:1950. doi:10.3389/fpls.2017.01950. 29204147 PMC5698779

[29] Siemiatkowska B, Chiara M, Badiger BG, Riboni M, D'Avila F, Braga D, Salem MAA, Martignago D, Colanero S, Galbiati M, et al. GIGANTEA is a negative regulator of abscisic acid transcriptional responses and sensitivity in Arabidopsis. Plant Cell Physiol. 2022;63(9):1285–1297. doi:10.1093/pcp/pcac102. 35859344

[30] Bensmihen S, Giraudat J, Parcy F. Characterization of three homologous basic leucine zipper transcription factors (bZIP) of the ABI5 family during Arabidopsis thaliana embryo maturation. J Exp Bot. 2005;56(412):597–603. doi:10.1093/jxb/eri050. 15642716

[31] Kim SY, Ma J, Perret P, Li Z, Thomas TL. Arabidopsis ABI5 subfamily members have distinct DNA-binding and transcriptional activities. Plant Physiol. 2002;130(2):688–697. doi:10.1104/pp.003566. 12376636 PMC166598

[32] Rivin CJ, Grudt T. Abscisic Acid and the developmental regulation of embryo storage proteins in maize. Plant Physiol. 1991;95(2):358–365. doi:10.1104/pp.95.2.358. 16667991 PMC1077538

[33] Riboni M, Robustelli Test A, Galbiati M, Tonelli C, Conti L. ABA-dependent control of GIGANTEA signalling enables drought escape via up-regulation of FLOWERING LOCUS T in Arabidopsis thaliana. J Exp Bot. 2016;67(22):6309–6322. doi:10.1093/jxb/erw384. 27733440 PMC5181575

[34] Zhao Y, Zhang Z, Gao J, Wang P, Hu T, Wang Z, Hou Y-J, Wan Y, Liu W, Xie S, et al. Arabidopsis duodecuple mutant of PYL ABA receptors reveals PYL repression of ABA-independent SnRK2 activity. Cell Rep. 2018;23(11):3340–3351 e5. doi:10.1016/j.celrep.2018.05.044. 29898403 PMC6085104

[35] Singh A, Jha SK, Bagri J, Pandey GK. ABA inducible rice protein phosphatase 2C confers ABA insensitivity and abiotic stress tolerance in Arabidopsis. PLoS One. 2015;10(4):e0125168. doi:10.1371/journal.pone.0125168. 25886365 PMC4401787

[36] Baek D, Shin G, Kim MC, Shen M, Lee SY, Yun D-J. Histone deacetylase HDA9 with ABI4 contributes to abscisic acid homeostasis in drought stress response. Front Plant Sci. 2020;11:143. doi:10.3389/fpls.2020.00143. 32158458 PMC7052305

[37] Verslues PE, Bray EA. Role of abscisic acid (ABA) and Arabidopsis thaliana ABA-insensitive loci in low water potential-induced ABA and proline accumulation. J Exp Bot. 2006;57(1):201–212. doi:10.1093/jxb/erj026. 16339784

[38] Maia J, Dekkers BJW, Dolle MJ, Ligterink W, Hilhorst HWM. Abscisic acid (ABA) sensitivity regulates desiccation tolerance in germinated A rabidopsis seeds. New Phytol. 2014;203(1):81–93. doi:10.1111/nph.12785. 24697728

[39] Ali A, Pardo JM, Yun DJ. Desensitization of ABA-signaling: the swing from activation to degradation. Front Plant Sci. 2020;11:379. doi:10.3389/fpls.2020.00379. 32391026 PMC7188955

[40] Shu K, Luo X, Meng Y, Yang W. Toward a molecular understanding of abscisic acid actions in floral transition. Plant Cell Physiol. 2018;59(2):215–221. doi:10.1093/pcp/pcy007. 29361058

